# Ipsilateral femoral neck and trochanter fracture

**DOI:** 10.4103/0019-5413.54765

**Published:** 2011

**Authors:** Devdatta S Neogi, K V Ajay Kumar, Vivek Trikha, Chandra Shekhar Yadav

**Affiliations:** Department of Orthopaedics, Jayprakash Narayan Apex Trauma Centre, All India Institute of Medical Sciences, Ansari Nagar, New Delhi, India

**Keywords:** Femoral neck fracture, neck-trochanter fracture, pertrochanteric fracture, dynamic condylar screw

## Abstract

Ipsilateral fractures in the neck and trochanteric region of the femur are very rare and seen in elderly osteoporotic patients. We present a case of a young man who presented with ipsilateral fracture of the femoral neck and a reverse oblique fracture in the trochanteric region following a motor vehicle accident. A possible mechanism, diagnostic challenge, and awareness required for identifying this injury are discussed.

## INTRODUCTION

Occurrence of simultaneous ipsilateral fracture of the femoral neck and trochanteric region is rare.^1^–^11^ Eleven cases are described in the English language literature and 65% of these have occurred in elderly with osteoporotic bones following a fall.^1^–^8^ We report a case of one such injury in a young polytrauma patient and review the challenges in the diagnosis and management of this injury.

## CASE REPORT

**Figure 1:**
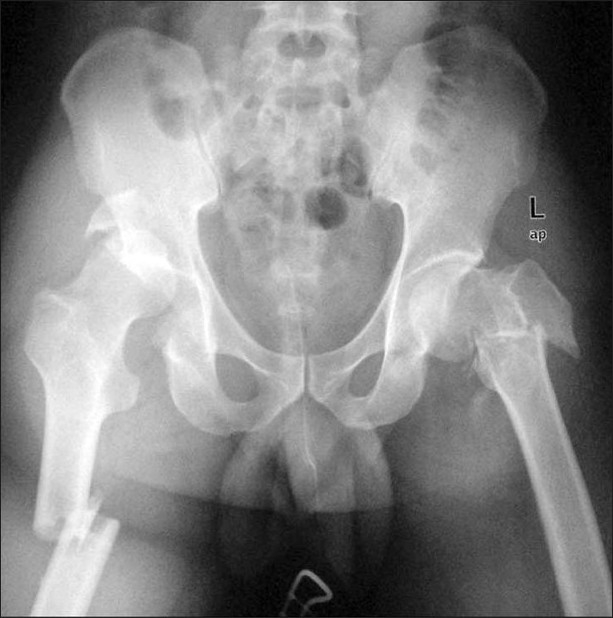
Radiograph of the pelvis including both hips and thighs (an anteroposterior view) at presentation showing pertrochanteric fracture of the left femur with an ipsilateral femoral neck fracture and posterior dislocation of the right hip with a posterior acetabular wall fracture with an ipsilateral right femoral shaft fracture

**Figure 2:**
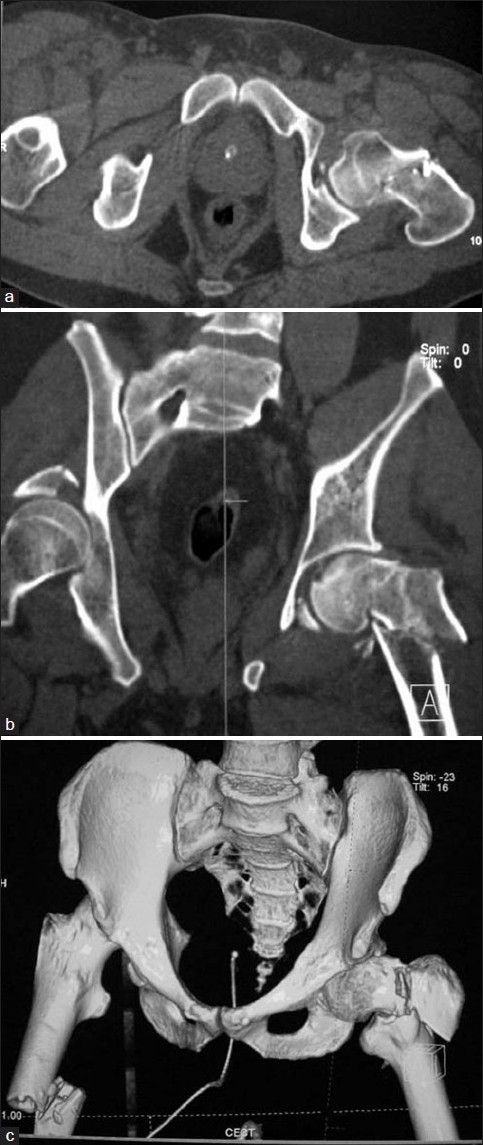
CT images of the pelvis including both hips. (a) A transverse section showing femoral neck fracture. (b) A section showing both femoral neck fracture and ipsilateral pertrochanteric fracture. (c) A 3D reconstruction image

A 28-year-old male patient was sitting next to the driver seat in a sports utility vehicle which met with road traffic accident. The sudden deceleration injury caused both the knees of the patient to hit against the dash board. The patient was taken to a level 3 trauma center and later shifted to our level 1 trauma center after 48 h of injury. On presentation, the patient was conscious, oriented and hemodynamically stable. The right thigh was deformed, painful, had abnormal mobility around thigh, and was more shortened compared to the left lower limb which was in an attitude of external rotation, there was contusion over the right gluteal region. The radiographs [Figure 1] revealed a posterior dislocation of the right hip with a posterior acetabular wall fracture, an ipsilateral fracture of the shaft of the femur, reverse oblique trochanteric fracture on the left side, and a both bones fracture of the right forearm. Being a high-energy injury and in order to study the acetabular fracture morphology in detail, a noncontrast computed tomography (CT) scan with a 3D reconstruction of the pelvis was done which revealed acetabular fracture geometry and a minimally displaced femoral neck fracture on the left side [Figure 2 a–c]. On a careful review of previous radiographs, a suspicious fracture line was seen on the left side, though it was not very clear.

**Figure 3:**
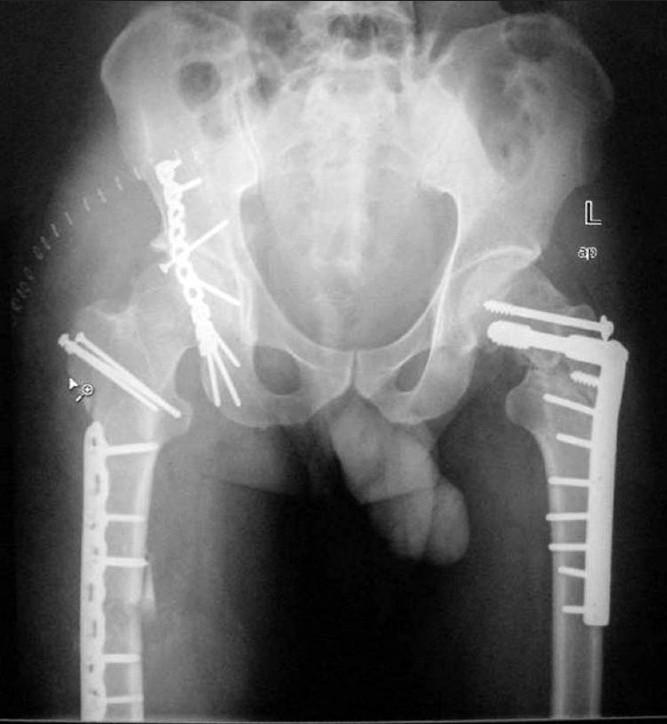
Postoperative radiograph of the pelvis with both hips and thighs (an anteroposterior view) showing internal fixation of all the injuries

**Figure 4:**
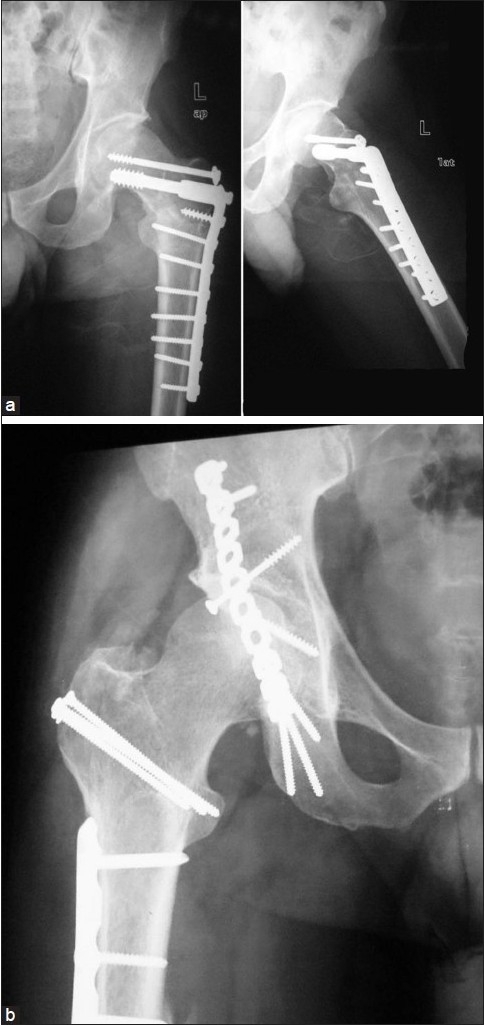
(a) Radiograph of the left hip with the thigh (an anteroposterior view) at 28 months showing a good union of both the fractures and no evidence of avascular necrosis. (b) Radiographs of the right hip with the thigh (an anteroposterior view) showing a good union at trochanteric osteotomy and acetabular fracture with no evidence of avascular necrosis

**Figure 5:**
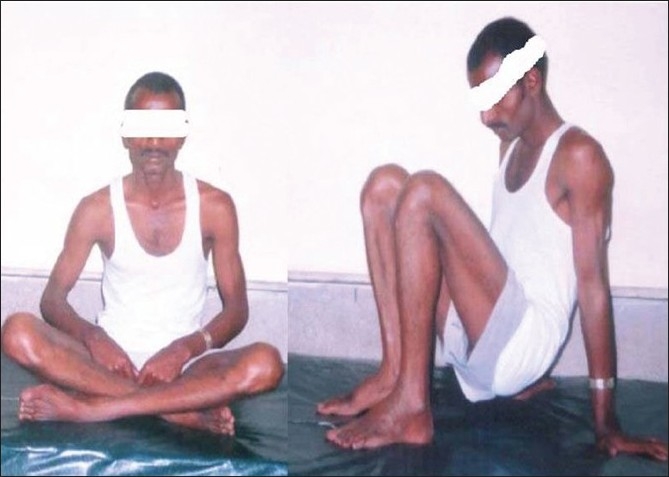
Clinical photograph of the patient at 28-months follow-up showing the range of motion at both the hip joints

The patient underwent open reduction and internal fixation of all his fractures in the same sitting having an irreducible dislocation on one side and a femoral neck fracture on the other about 50 h from his time of sustaining injury. First with the patient in left lateral position on the right side, open reduction and internal fixation (ORIF) of the femoral shaft fracture with a locking plate and of the posterior wall acetabular fracture with a reconstruction plate and lag screws was done. A trochanteric osteotomy was also performed on the right side to aid in the fixation of the high posterosuperior acetabular wall fracture and the osteotomy subsequently fixed with three 3.5 mm cortical screws with washers. Then the patient was put over a fracture table and the ipsilateral fracture of the femoral neck and pertrochanteric fracture on the left side were treated with a dynamic condylar screw (DCS) and an additional cannulated cancellous screw [Figure 3]. During fluoroscopy, care was taken to ensure that all the screw threads crossed the fracture lines and compression was obtained at the neck region. Finally, the fracture of the both bones of the right forearm was fixed with low-contact dynamic compression plates (LC-DCP). It took around 6 hours for all the procedures to be completed with a blood loss of 1.2 liters and patient received 3 units of blood transfusion. Postoperatively, the patient had in bed mobilization from the second postoperative day, and started weight bearing with crutches at 12 weeks. All fractures united by 5 months, and at 28-month follow-up, the patient has no evidence of avascular necrosis, and an excellent functional outcome [Figures 4 and 5].

## DISCUSSION

There are 11 reports in the medical literature of ipsilateral fractures of the femoral neck and intertrochanteric--pertrochanteric region.^1^–^11^ Of these, eight cases are reported in elderly osteoporotic patients following a fall.^1^–^8^ One case was of a 54-year-old person caught in olive press^11^ and two cases were of patients with a motor vehicle accident.^9^,^10^ The index case also followed a motor vehicle accident in a young adult male. We postulate that the patient sustained a dashboard injury which initially caused the reverse oblique trochanteric fracture, and the continued movement of the distal fragment impacted the femoral neck and caused the femoral neck fracture which was minimally displaced.

This injury being rarely seen can easily be missed on radiographic evaluation. Of the cases reported in the literature, five cases were apparent at initial radiographic evaluation,^4^,^6^,^8^–^10^ three were confirmed on further imaging preoperatively,^3^,^5^,^7^ two were identified by fluoroscopy during surgical procedure,^1^,^2^ while one was identified in a postoperative period.^11^ In our case, the preliminary examination of the anteroposterior radiograph did not reveal the presence of a fracture line in the femoral neck region. A lateral radiograph was not obtained in view of the multiple injuries that he had sustained. A CT scan with a 3D reconstruction of the pelvis performed for the evaluation of the contralateral acetabular fracture with a dislocation of hip revealed a fracture line at the femoral neck on the left side. Thus a CT scan with 3D reformatting was helpful in preoperative diagnosis of this injury.

A prosthetic replacement may be an option in the management of such injuries in elderly patients; however, in a young adult, preserving the femoral head should be the goal.^9^,^10^ The presence of this combination injury presents additional technical difficulties in attempted osteosynthesis.^10^ In one of the cases, the subcapital femoral neck fracture was missed preoperatively and during the insertion of the DHS screw, rotation of the femoral head might have occurred with an interruption in blood supply resulting later in avascular necrosis.^11^ A sliding hip screw is the most commonly used implant for the fixation of intertrochanteric fractures.^12^ However, many investigators^12^,^13^ have reported that this device is not suitable for 31-A3 reverse oblique or transverse fractures and a DCS or an intramedullary nail may be a better device.^12^,^13^ In the presence of an additional femoral neck fracture, the use of an im nail to fix this combination injury is not only a technically demanding procedure, but also there is no literature support on its use in a femoral neck fracture. Our contention also at that time was that an im nail may displace the neck fracture more and we may not get sufficient compression at the fracture site. Another important concern is the larger proximal diameter of these implants which may require the reaming of the trochanter to 15–18 mm.^14^ The long-term importance of removing this amount of bone from the proximal femur in a young patient is unknown^14^ and should be considered cautiously more so in the case of a combination fracture. Hence, in this patient having multiple fractures we performed closed reduction under fluoroscopy and fixation with DCS with an additional derotation screw. This 16-mm cancellous cannulated derotation screw also helped in achieving additional compression at the femoral neck fracture site. In the cases reported earlier [Table 1] dynamic hip screw (DHS) with or without supplemental fixation was used in six cases,^2^,^4^,^5^,^8^,^9^,^11^ *in situ* pinning,^3^ hemiarthroplasty alone,^7^ hemiarthroplasty with Parham bands,^1^ percutaneous compression plate (PCCP)^6^ in one case each, and cancellous cannulated screws, Knowles pin and dynamic compression plate (DCP) in one.^10^

**Table 1:** Review of the literature

Author/ year	Age/ sex	Mechanism	Fracture type	Diagnosis	Implant used	Follow-up	Outcome
An, 1989^1^	NA	Low-energy fall	Intertrochanteric + neck	At surgery	Hemiarthroplasty with Parham bands	NA	Good
Cohen, 1999^2^	79/F	Low-energy fall	Pertrochantercic + subcapital	At surgery	DHS	24 months	Ambulate cane, no AVN
Lawrence, 1993^3^	NA	Low energy fall	Intertrochanteric and subcapital	Preop. (+)	Pinning		Patient died; death not related to surgery
Kumar, 2001^4^	83/F	Low energy fall	Intertrochanteric + subcapital	Preop.	DHS + TSP + ARS	12 months	Pain-free ambulation, partial head collapse with AVN
Pemberton, 1989^5^	73/F	Low energy fall	Subcapital + basicervical	Preop. (+)	DHS	30 months	Good, no AVN
Poulter, 2007^6^	76/F	Low energy fall	Sub capital+ intertrochanteric	Preop.	PCCP	4 months	Good
Yuzo, 2001^7^	89/F	Low energy fall	Neck + trochanter	Preop. (+)	Bipolar prosthesis	NA	Good
Sayegh 2005^8^	54/M	Olive press	Per-trochantercic + subcapital	Preop.	DHS + cerclage wire	58 months	Good, no AVN, 2 cm short
Butt, 2007^9^	30/M	RTA	Neck + reverse oblique	Preop.	DHS + ARS	12 months	Good, no AVN
Dhar, 2008^10^	30/M	RTA	T-shaped	Preop.	DCP + lag screws	12 months	Good, no AVN
Perry, 2008^11^	86/F	Low energy fall	Intertrochanteric + neck	Post-op	DHS	12 weeks	Early AVN, failure, refused THR
Neogi, (present case)	28/M	RTA	Neck + pertrochanteric	Preop. (+)	DCS + ARS	28 months	Good, no AVN

RTA = road traffic accident; (+) = additional imaging investigations needed to diagnose; DHS = dynamic hip screw; DCS = dynamic condylar screw; ARS = antirotation screw; PCCP = percutaneous compression plating; AVN = avascular necrosis.

Of the six cases fixed with DHS, successful result was present in five cases while one case^11^ in whom fracture was recognized postoperatively had fixation failure. One case^3^ with pinning in situ died from complications not related to surgery. Good result was also seen with hemiarthroplasty with or without Parham bands.^1^,^7^ At 4-month follow-up, the case who had a fixation with PCCP^6^ had a good result. The final case with cancellous cannulated screws, Knowles pin, and DCP^10^ also had good result at 1 year. Both the fractures united in our case and at 28-month follow-up, the patient has no signs of avascular necrosis and a nearly painless full range of motion at hip and knee and ambulates without any aids.

## References

[1] An HS, Wojcieszek JM, Cooke RF, Limbird R, Jackson WT (1989). Simultaneous ipsilateral intertrochanteric and subcapital fracture of the hip. A case report. Orthopedics.

[2] Cohen I, Rzetelny V (1999). Simultaneous ipsilateral pertrochanteric and subcapital fractures. Orthopaedics.

[3] Lawrence B, Isaacs C (1993). Concomitant ipsilateral intertrochanteric and subcapital fracture of the hip. J Orthop Trauma.

[4] Kumar R, Khan R, Moholkar K, Smyth H, Borton D (2001). A rare combination fracture of the neck of femur. Eur J Orthop Surg Traumatol.

[5] Pemberton DJ, Kriebich DN, Moran CG (1989). Segmental fracture of the neck of the femur. Injury.

[6] Poulter RJ, Ashworth MJ (2007). Concomitant ipsilateral subcapital and intertrochanteric fractures of the femur. Injury Ext.

[7] Yuzo O, Yamanaka M, Hiroshi T, Naoyoshi I (2001). A case of femoral neck and trochanteric fracture in ipsilateral femur. Orthop Traumatol.

[8] Sayegh F, Karataglis D, Trapotsis S, Christopforides J, Pournaras J (2005). Concomitant ipsilateral pertrochanteric and subcapital fracture of the proximal femur. Eur J Trauma.

[9] Butt MF, Dhar SA, Hussain A, Gani NU, Kangoo KA, Farooq M (2007). Femoral neck fracture with ipsilateral trochanteric fracture: Is there room for osteosynthesis?. Internet J Orthop Surg [Internet].

[10] Dhar SA, Mir MR, Butt MF, Farooq M, Ali MF (2008). Osteosynthesis for a T-shaped fracture of the femoral neck and trochanter: A case report. J Orthop Surg.

[11] Perry DC, Scott SJ (2008). Concomitant ipsilateral intracapsular and extracapsular femoral neck fracture: a case report. J Med Case Reports.

[12] Haidukewych GJ, Israel TA, Berry DJ (2001). Reverse obliquity fractures of the inter-trochanteric region of the femur. J Bone Joint Surg Am.

[13] Sadowski C, Lübbeke A, Saudan M, Riand N, Stern R, Hoffmeyer P (2002). Treatment of reverse oblique and transverse intertrochanteric fractures with use of an intramedullary nail or a 95° screw-plate: A prospective, randomized study. J Bone Joint Surg Am.

[14] Sims SH (2002). Subtrochanteric femoral fractures. Orthop Clin N Am.

